# The frontal association area: exercise-induced brain plasticity in children and adolescents and implications for cognitive intervention practice

**DOI:** 10.3389/fnhum.2024.1418803

**Published:** 2024-09-05

**Authors:** Ziyun Zhang, Peng Shi, Kai Zhang, Chenyang Li, Xiaosu Feng

**Affiliations:** ^1^School of Life and Health, Huzhou College, Huzhou, China; ^2^School of Physical Education, Shanghai University of Sport, Shanghai, China; ^3^School of Physical Education, Shandong University of Aeronautics, Binzhou, China; ^4^Department of Graduate Studies, Shenyang Sport University, Shenyang, China; ^5^Department of Physical Education, Huaiyin Institute of Technology, Huaian, China; ^6^School of Physical Education, Liaoning Normal University, Dalian, China

**Keywords:** frontal association area, brain, neuroplasticity, fMRI, exercise, ALE meta-analysis

## Abstract

**Objective:**

Explore the plasticity of the frontal associative areas in children and adolescents induced by exercise and potential moderating variables.

**Methods:**

Computer searches of CNKI, WOS, PubMed and EBSCO databases were conducted, and statistical analyses were performed based on SPSS 25.0, Stata 12.0 and Ginger ALE 2.3 software after literature screening, data extraction and quality assessment were performed independently by two researchers.

**Results:**

A total of 13 articles, including 425 participants aged 8.9∼16.8 years, were included. Frequency analysis revealed that exercise induced enhanced activation in frontal, parietal, occipital, limbic system and cerebellum (*P* < 0.01). Activation Likelihood Estimation (ALE) meta-analysis revealed that exercise altered the activation status of the frontal association (medial frontal gyrus, middle frontal gyrus, inferior frontal gyrus and precentral gyrus), cuneus, lingual gyrus, cingulate gyrus, parahippocampal gyrus, caudate nucleus and cerebellar apex, with the volume of activation in the frontal association accounting for 61.81% of the total activation cluster volume and an enhanced activation effect. Additionally, the study design, age, gender, nationality, cognitive tasks, as well as exercise intensity, intervention time, and type of exercise may be potential moderating variables. Particularly, sustained exercise induced a decrease in activation in the left parahippocampal gyrus, culmen, and lingual gyrus, while variable exercise induced an increase in activation in the left middle frontal gyrus.

**Conclusion:**

Exercise-induced activation increase in the frontal associative areas of children and adolescents is dominant, especially longer periods of moderate-intensity variable exercise can induce more brain region activation. However, some of the included studies are cross-sectional, and the accuracy of the results still requires further verification.

**Systematic review registration:**

https://www.crd.york.ac.uk/prospero/, identifier PROSPERO, CRD42022348781.

## 1 Introduction

The human brain is an extremely complex functional system that controls all thoughts and actions of the individual. The frontal association area is the higher management center for the formation and control of various behaviors and is closely associated with higher cognitive functions such as executive control, working memory, logical reasoning and fluid intelligence (Carlén, 2017; Eimontaite et al., 2018; Kane and Engle, 2002; Welsh et al., 1991). Additionally, the higher-order literacies of children and adolescents, such as logical thinking, analysis, synthesis, reasoning, deduction, induction, and hypothesis (Cai, 2014), as well as their autonomous and conscious actions and complex communication (Teng, 2016), are all regulated by the frontal association area. Based on this, Pan (2018) proposed a hypothesis that the development of students’ advanced literacy skills could be promoted in accordance with the developmental laws of the prefrontal cortex. Against this backdrop, how to promote the plasticity of the frontal association area in children and adolescents has become a focal point of research in various fields.

A series of studies have confirmed that exercise can induce neuroplasticity in the prefrontal association area, thereby improving cognitive performance. For example, Activation Likelihood Estimation (ALE) meta-analyses have shown that exercise induces significant activation of prefrontal brain areas such as the superior frontal gyrus (Wu et al., 2022) and inferior frontal gyrus (Cui et al., 2019) in executive control task activities. Changes in brain activation states induced by exercise are common and possess temporal and spatial characteristics (Cui et al., 2019; Li and Zhang, 2023). The temporal feature refers to the changes in brain activation states before and after exercise intervention. The spatial feature refers to the changes in the spatial location of brain activation after exercise intervention, mainly including three patterns: activation enhancement, activation reduction, and activation re-organized (Cui et al., 2019). Activation enhancement refers to the increase in the activation volume of relevant brain regions after the intervention. Activation reduction refers to the decrease in the activation volume of relevant brain regions after the intervention. Activation re-organized refers to the phenomenon where after the intervention, the activation volume of relevant brain regions coexists with both enhancement and reduction, resulting in a new layout. The spatio-temporal characteristics of exercise-induced neuroplasticity can be explained by the Extended Frontoparietal Integration Theory (Gur et al., 2021) and the Neural Efficiency Hypothesis (Chen et al., 2022). The former considers cognitive and thinking activities to be the result of the synergistic action of various brain areas, including the frontal, parietal, temporal, occipital, limbic system, striatum and cerebellum (Gur et al., 2021; Jung and Haier, 2007). The latter is more supportive of the idea that “smart brains are lazy”, that is, that individuals use fewer neural resources to complete cognitive tasks (Chen et al., 2022). Based on the central role of the frontal association area in cognitive tasks and previous studies (Chen et al., 2022; Li and Zhang, 2023), this hypothesis can be derived: Exercise induces a reorganization of activation in a series of brain regions, with a more significant increase in activation in the frontal association area. However, this hypothesis still requires further verification and argumentation.

In addition, after a comprehensive review of the relevant studies in this field, several important limitations have indeed been identified. Firstly, there are significant inconsistencies or even complete contradictions in the results of different studies regarding the impact of exercise on brain plasticity in children and adolescents. For instance, Krafft et al. (2014) found that after exercise intervention, children showed increased activation in the frontal medial gyrus, frontal middle gyrus, and other frontal lobe regions during the Flanker task. However, Herting and Nagel (2013) discovered that children with high aerobic fitness generally showed decreased activation in brain regions during verbal associative memory encoding tasks. Secondly, while there have been systematic reviews (Meijer et al., 2020; Wassenaar et al., 2020; Valkenborghs et al., 2019) studying the effects of exercise on brain plasticity in children and adolescents, the conclusions drawn from these reviews need to be treated with caution as they are primarily based on qualitative analysis. Therefore, there is still a need for more rigorous quantitative methods such as ALE meta-analysis to assess changes in brain activation states. However, there is still a scarcity of relevant research on children and adolescents, limiting our in-depth understanding of the changes in brain plasticity in this specific group. Lastly, although Li and Zhang (2023) have recognized the importance of quantitatively integrating research findings, they have not yet fully taken into account potential moderating factors, thereby overlooking the heterogeneity that may exist.

Differences in study design, sample characteristics, exercise characteristics, and task paradigms may be the reasons for inconsistencies among original studies. Specifically, the characteristics of exercise type, intensity, and period can have significantly different cognitive benefits on the human brain (Heilmann et al., 2022; Liu, 2017). Regarding the type of exercise, exercises that induce more cognitive challenges are more likely to produce greater cognitive advantages compared to continuous aerobic exercise (Shi and Feng, 2022; Shi et al., 2022). Based on this, Shi et al. (2023) categorized exercise types into variable exercise and sustained exercise, and called on researchers to explore the differences in cognitive benefits and brain plasticity resulting from these two types of exercise. Variable exercise refers to exercise types with relatively complex movements and action structures, which mainly promote perceptual-motor skills, agility, and coordination (Mansouri et al., 2009; Pesce et al., 2019). Examples include open skills like basketball and football, as well as closed-sequential skills like aerobics and martial arts. Sustained exercise, on the other hand, refers to exercise types with relatively simple movement structures and actions without clear beginnings or ends, which mainly develop cardiorespiratory fitness (Zhu et al., 2014). These include activities like running, swimming, and cycling. Based on this, this study focuses on exploring the potential role of exercise types while investigating potential moderating factors, and puts forward the hypothesis that there exists heterogeneity in the brain activation induced by variable exercise and sustained exercise.

This study aims to systematically review research on brain activation induced by exercise in children and adolescents, and to explore whether the brain plasticity induced by exercise conforms to the Extended Frontoparietal Integration Theory and the Neural Efficiency Hypothesis using ALE meta-analysis. On this basis, this study further aims to investigate the moderating variables of exercise-induced brain plasticity and to explore the optimal set of elements that promote brain plasticity. Through this study, we hope to provide theoretical and practical references for research related to promoting brain health and cognitive functions in children and adolescents through exercise, and to offer guidance for neuroscientific- based physical education practices.

## 2 Materials and methods

This ALE meta-analysis was registered (CRD42022348781) in the International Prospective Register of Systematic Reviews (PROSPERO). The Preferred Reporting Items for Systematic Reviews and Meta-Analyses (PRISMA 2020) guidelines were followed for this study.

### 2.1 Search strategies

A search of the relevant articles was conducted by one researcher using both English and Chinese search terms. This study employs the following search strings for literature retrieval in the CNKI, Web of Science (WOS), PubMed, and EBSCO databases. The example of a search string in the WOS database is as follows: (TS = (((exercise OR sports OR fitness OR physical activity) AND (magnetic resonance OR brain imaging OR brain function OR brain activation OR functional MRI OR fMRI) AND (children OR child OR adolescents OR young OR teenagers OR kindergarten)))) NOT TS = ((concussion OR brain injury OR injury)). In addition, recent systematic reviews and Meta-analyses were searched as well as references back to included studies to avoid omissions. The search time frame is from the creation of this database to June 2024.

### 2.2 Selection criteria

This study designed the inclusion and exclusion criteria for the literature according to the Population, Intervention, Comparison, Outcomes and Study (PICOS) principles (Costantino et al., 2015). Inclusion criteria: (1) subjects were typical children and adolescents; (2) interventions or exposures were acute or long-term exercise; (3) controls included routine academic life, attention and behavioral control, irregular exercise and sedentary; (4) outcome variables included functional magnetic resonance imaging (fMRI) tasks and brain activation results; (5) study designs included randomized controlled trial (RCT), quasi-experimental design (QED) and cross- sectional study (CSS). Exclusion criteria: (1) special groups such as athletes, pilots, police, military, and atypical groups with cognitive or intellectual impairment; (2) comprehensive interventions such as exercise combined with diet control, exercise combined with cognitive therapy; (3) studies in which detailed information is not available such as reviews, abstracts, letters, comments; (4) resting-state MRI studies; (5) repeated publications on the same study subjects, including only those of high quality; (6) literature with missing or incomplete data (MNI or Talairach coordinates) in the literature. Selection was carried out independently by two researchers, with the other two researchers carrying out a secondary assessment of the screened articles, and where there was controversial article, the group discussed and agreed together.

### 2.3 Data extraction

By thoroughly reading the included literature, the following data are extracted: (1) bibliographic information, including the first author and the year of publication; (2) study design, including RCT, QED, or CSS; (3) basic information of participants, including sample size, age, proportion of female participants, and nationality; (4) intervention or exposure measures, including exercise methods (categorized into variable exercise and sustained exercise based on exercise type), exercise intensity (low, moderate, and high), exercise duration (acute and long-term), frequency, single time, and total intervention time (calculated based on the formula “duration × frequency × single time”); (5) control measures; (6) cognitive task paradigms used during fMRI, such as inhibitory control tasks like Flanker, GO/NO GO, and working memory tasks like N-back; (7) brain coordinate positioning systems adopted in the research, including MNI and Talairach; (8) brain activation areas and their activation coordinates. In this study, the extracts were entered into Excel 2010 and saved. The data extraction is carried out independently by two researchers and the extraction is secondarily assessed by the other two researchers, and if there is a dispute, the group discusses and decides together.

### 2.4 Quality assessment

This quality assessment of RCTs was performed using the Cochrane Collaboration Risk of Bias Tool (Cumpston et al., 2019). The tool assesses six aspects of randomized methods, blinding, allocation concealment, completeness of outcome data, selective reporting of study results and other biases. The Methodological Index for Non-Randomized Studies (MINORS) scale (Slim et al., 2003) was used to assess the quality of QEDs. The tool consists of 12 items, with 9 to 12 additional criteria for assessing studies with a control group, each with a score of 2, for a total score of 24. A score of 0 means not reported; a score of 1 means reported but with insufficient information; a score of 2 means reported and sufficient information provided. The Agency for Healthcare Research and Quality (AHRQ) scale (Bindman, 2017) was used to assess the quality of CSSs. The tool consists of 11 items and the assessor is asked to assess each item using “yes”, “no” and “unclear”. Given that the CSS were all human experiments and there was no follow-up, items 4 and 9 were excluded and only 9 items were assessed for quality. Judgments based on the assessment tool were made independently by two researchers and, where there was significant disagreement, the items were discussed with a third researcher.

### 2.5 Data analysis

Firstly, this study was based on SPSS 25.0 and Stata 12.0 software, using frequency analysis and χ^2^ tests to explore exercise-induced increased and decreased activation in brain areas. In addition, this study employs regression analysis to investigate the association between the total intervention time and the number of activated brain regions. Secondly, this study used ALE meta-analysis to examine the brain activation state reflected by exercise-induced fMRI data. Based on Ginger ALE 2.3 software for ALE meta-analysis under the Talairach spatial standard, Lancaster was used to convert the coordinates reported in the MNI spatial standard to Talairach coordinates (Lancaster et al., 2007). Referring to the algorithm and parameter setting recommendations provided in the Ginger ALE manual, the FDR pID algorithm was chosen for this study, with a critical statistical value of 0.001 and a minimum volume setting of 200 mm (Eickhoff et al., 2009), to calculate exercise-induced brain activation clusters and their maximum ALE values (maximum ALE values indicate the probability of activation of brain areas). Thirdly, based on Mango 4.0 software and referring to previous study (Wu et al., 2022), the activation coordinates of brain regions from the ALE meta-analysis were superimposed onto a standard brain image (Colin_tlrc_2x2x2 from )^1^ to visualize the results of this study.

Finally, to verify whether the ALE results overlap with the frontal association area or other brain networks, this study adopted the method of Meta-Analytic Connectivity Modeling (MACM), following the procedure implemented by Robinson et al. (2010) in NeuroSynth.^2^ This study used activation clusters as the regions of interest (ROI) derived from the ALE results and input them into the NeuroSynth program to evaluate MACM. The program searched for studies reporting activation within spherical seeds (6mm) around those coordinates in over 11,000 fMRI studies, and then combined the identified co-activations to form the MACM output (Wu et al., 2022). Additionally, the program assigned a *Z*-score to each voxel, indicating the strength of the association between the given voxel and the seed coordinates.

## 3 Results

### 3.1 Selection results

**FIGURE 1:**
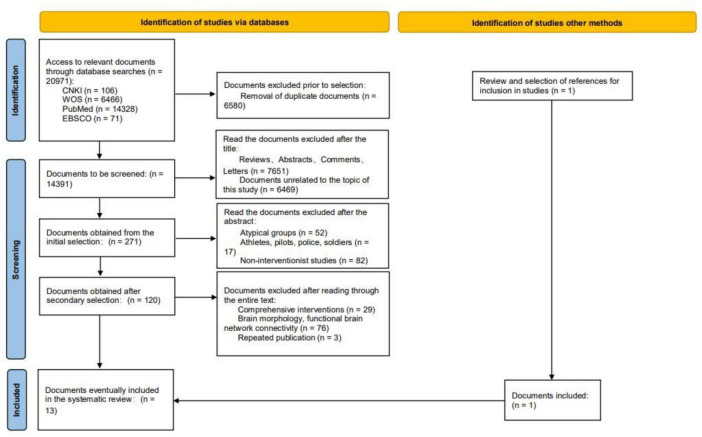
Flow chart for literature selection.

In this study, 20,971 articles were retrieved and the retrieved articles were imported into EndNote X9 software for de-duplication, resulting in 14,391 articles. A total of 13 articles were selected for inclusion. The literature selection process is shown in Figure 1.

### 3.2 Data extraction results

Thirteen articles were published between 2011 and 2024, including 5 (38.46%) RCTs, 5 (38.46%) QEDs, and 3 (23.08%) CSSs. These 13 articles included a total of 425 participants with an average age ranging from 8.9 to 16.8 years old. Except for two studies (Jin, 2016; Qu et al., 2024), the remaining studies reported the gender of the participants. Among them, Herting and Nagel (2013) specifically explored the characteristics of brain activation in girls, while the proportion of girls in other studies ranged from 44.4% to 71.0%. The participants mainly came from the United States (Chaddock et al., 2012; Chaddock-Heyman et al., 2013; Davis et al., 2011; Herting and Nagel, 2013; Krafft et al., 2014; Sachs et al., 2017; Voss et al., 2011), China (Chen et al., 2011; Chen et al., 2016b; Jin, 2016; Qu et al., 2024; Zhu et al., 2021), and Canada (Metcalfe et al., 2016).

The included intervention studies mainly comprise acute interventions (Chen et al., 2011; Chen et al., 2016b; Metcalfe et al., 2016) and long-term interventions (Chaddock-Heyman et al., 2013; Davis et al., 2011; Jin, 2016; Krafft et al., 2014; Qu et al., 2024; Sachs et al., 2017; Zhu et al., 2021). Among them, five studies (Chen et al., 2011; Chen et al., 2016b; Jin, 2016; Qu et al., 2024; Zhu et al., 2021) utilized moderate-intensity interventions, two studies (Davis et al., 2011; Krafft et al., 2014) applied high-intensity interventions, and two studies (Chaddock-Heyman et al., 2013; Metcalfe et al., 2016) adopted moderate-to- high-intensity interventions. The duration of acute interventions ranged from 27 to 30 minutes, while the duration of long-term interventions varied from 11 weeks to 2 years, with a total intervention time spanning from 520 to 31,200 minutes. Five studies (Chaddock-Heyman et al., 2013; Chen et al., 2011; Chen et al., 2016b; Krafft et al., 2014; Metcalfe et al., 2016) employed sustained exercise interventions aimed at improving cardiorespiratory health and aerobic fitness, while five studies (Davis et al., 2011; Jin, 2016; Qu et al., 2024; Sachs et al., 2017; Zhu et al., 2021) utilized variable exercise interventions focused on learning complex motor skills and enhancing agility and coordination. In addition, the included CSSs mainly categorized participants into high and low aerobic fitness groups based on maximal oxygen consumption (Chaddock et al., 2012; Voss et al., 2011) or the duration of aerobic exercise (Herting and Nagel, 2013).

FMRI tasks are mainly divided into three categories: inhibitory control, working memory, and memory encoding. Among them, the inhibitory control task paradigms adopted by researchers include Flanker, Antisaccade, GO/NO GO, and Stroop; the working memory task paradigm adopted by researchers is N-back; and the memory encoding task is Lexical associative memory encoding. Seven studies (53.85%) showed increased brain activation among participants; four studies (30.77%) showed decreased brain activation; and three studies (23.08%) showed re-organized of brain activation after the intervention. More detailed basic information on the included articles can be found in Table 1; for detailed results of the brain activation changes, please refer to Supplementary Material 1; the narrative review included in the study is attached in Supplementary Material 2.

**TABLE 1:** Basic characteristics of the included studies.

Included studies	Study design	Subject characteristics				Interventions/exposures and controls	Main results	
		**Sample size**	**Age**	**Girls(%)**	**Nationality**		**Tasks (results)**	**Brain activation**
Chen et al., 2011	QED	9	10	55.6%	CHN	One 30 min moderate intensity (60∼69% HRmax) power bike (SE) intervention vs. pre-intervention	Flanker (↑)	Re-organized (MNI)
Davis et al., 2011	RCT	E = 56 C = 60	7∼11	56.0%	USA	13 ± 1.6 weeks high intensity (166 ± 8 bpm) exercise (running games, jump rope, and modified basketball and soccer) (VE), 2 times/ week, 20 min/time (520 min) vs. sedentary activities	Antisaccade (↑)	Re-organized (Talairach)
Voss et al., 2011	CSS	E = 18 C = 18	E = 9.8 ± 0.6 C = 9.9 ± 0.6	E = 55.6% C = 38.9%	USA	Higher aerobic group, 53.6 ± 5.6 VO_2_ max vs. lower aerobic group, 37.0 ± 4.1 VO_2_max	Flanker (↑)	Re-organized (MNI)
Chaddock et al., 2012	CSS	E = 14 C = 18	E = 9.7 ± 0.6 C = 10.1 ± 0.5	E = 64.3% C = 38.9%	USA	Higher aerobic group, 53.1 ± 4.5 VO_2_ max vs. lower aerobic group, 36.6 ± 4.4 VO_2_max	Flanker (↑)	Increased (MNI)
Chaddock-Heyman et al., 2013	RCT	E = 14 C = 9	E = 8.9 ± 0.7 C = 8.9 ± 0.4	E = 50.0% C = 66.7%	USA	36 weeks of moderate to high intensity intervention to improve cardiorespiratory fitness, muscle strength, and motor skills (SE), 5 times/week, 60min/time (10800min) vs. wait control	GO/NO GO (↑)	Decreased (MNI)
Herting and Nagel, 2013	CSS	E = 17 C = 17	E = 16.6 ± 0.8 C = 16.2 ± 0.8	E = 100.0% C = 100.0%	USA	Higher fitness group, ≥ 10h/week aerobic activities vs. lower fitness group, ≤ 1.5h/week aerobic activities	Lexical associative memory encoding (→)	Decreased (Talairach)
Krafft et al., 2014	RCT	E = 22 C = 17	E = 9.7 ± 0.8 C = 9.9 ± 0.9	E = 71.0% C = 58.0%	USA	32 weeks high (161 ± 9 bpm) aerobic exercise (SE), 7 times/week, 40min/time (8960min) vs. sedentary attention control	Antisaccade (↑) Flanker (↑)	Decreased (MNI) Increased (MNI)
Jin, 2016	QED	E = 14 C = 12	E = 10.1 ± 1.0 C = 11.5 ± 0.8	NA	CHN	11 weeks moderate intensity (60∼69% HR max) combined exercise program (fancy running ++ martial arts + fancy jumping rope) (VE), 4 times/week, 30min/time (1320min) vs. regular activities	2-back (↑)	Increased (MNI)
Chen et al., 2016b	QED	9	10	44.4%	CHN	One 30 min moderate intensity (60∼69% HRmax) power bike (SE) intervention vs. pre-intervention	2-back (↑)	Increased (MNI)
Metcalfe et al., 2016	QED	30	16.8 ± 1.4	57.0%	CAN	One 27min moderate to high (60∼80% HRmax) power bike (SE) intervention vs. pre-intervention	GO/NO GO (→)	Decreased (MNI)
Sachs et al., 2017	QED	E = 13 C = 17	E = 8.9 ± 0.6 C = 9.1 ± 0.5	E = 55.6% C = 4.0%	USA	2 years soccer (3 times/week) and swimming (2 times/week) exercise, 60min/time (VE) (31200 min) vs. daily activities	Stroop (↑)	Increased (MNI)
Zhu et al., 2021	RCT	E = 9 C = 8	E = 11.2 ± 1.2 C = 11.8 ± 0.9	E = 44.4% C = 50.0%	CHN	11 weeks moderate intensity (60–69% HR max) after-school exercise (running games, jump rope, martial arts) (VE), 4 times/week, 30min/time (1320 min) vs. no additional exercise	N-back (↑)	Increased (MNI)
Qu et al., 2024	RCT	E = 12 C = 12	NA	NA	CHN	11 weeks moderate intensity (60–69% HR max) aerobic exercise (fancy running + martial arts + fancy jumping rope) (VE) vs. regular activities	2-back (↑)	Increased (MNI)

E, experimental/exposure group; C, control group; NA, not applicable; RCT, randomized controlled trial; QED, quasi- experimental design; CSS, cross-sectional study; CHN, China; USA, United States; CAN, Canada; VE, variable exercise; SE, sustained exercise; HRmax, maximum heart rate; bpm, heartbeat rhythm; VO2max, maximum oxygen uptake; ↑ indicates improved cognitive task performance in the intervention/exposure group compared with the control group; → indicates that cognitive task performance was not significantly altered in the intervention/exposure group compared with the control group.

### 3.3 Quality assessment results

There was no bias in the 5 RCTs to selectively report study results, and it is unclear whether other biases existed. However, only one study (Davis et al., 2011) reported a blinded strategy, no study reported randomized methods and allocation concealment methods, and all outcome data were incomplete, with a lost access sample between 2 and 11 cases (Table 2). The 5 QEDs included two (40.0%) non-randomized controlled designs and three (60.0%) single-group pre and post-test designs, of which the former had a mean quality assessment score of 18 and the latter had a mean quality assessment score of 11. The main reasons for the lower quality were the failure to use an assessor-blinded strategy for endpoint indicator evaluation and the failure to estimate the sample size (Table 3). All 3 CSSs reported on sources, subject selection criteria, quality assessment strategies, and control of confounders. However, it is not clear what time stage the subjects were identified, the subjective factors of confounding by the assessors, the explanation and manipulation of missing data, and the response rate of the participants (Table 4).

**TABLE 2:** RCT quality assessment results.

Included studies	Randomized methods	Blinding	Allocation concealment	Completeness of outcome data	Selective reporting of study results	Other biases
Davis et al., 2011	Unclear	Double-blind	Unclear	7 cases of missed visits	No	Unclear
Chaddock-Heyman et al., 2013	Unclear	Unclear	Unclear	9 cases of missed visits	No	Unclear
Krafft et al., 2014	Unclear	Unclear	Unclear	11 cases of missed visits	No	Unclear
Zhu et al., 2021	Unclear	Unclear	Unclear	9 cases of missed visits	No	Unclear
Qu et al., 2024	Unclear	Unclear	Unclear	2 cases of missed visits	No	Unclear

**TABLE 3:** QED quality assessment results.

Included studies	(1)	(2)	(3)	(4)	(5)	(6)	(7)	(8)	(9)	(10)	(11)	(12)	Total
Chen et al., 2011	2	2	2	2	0	1	2	0	NA	NA	NA	NA	11
Jin, 2016	2	2	2	2	0	1	2	0	2	2	2	2	19
Chen et al., 2016b	2	2	2	2	0	1	2	0	NA	NA	NA	NA	11
Metcalfe et al., 2016	2	2	2	2	0	1	2	0	NA	NA	NA	NA	11
Sachs et al., 2017	2	2	2	2	0	1	0	0	2	2	2	2	17

(1) the purpose of the study was clearly given; (2) the consistency of the included subjects; (3) the expected data collection; (4) the outcome indicators appropriately reflected the purpose of the study; (5) the objectivity of the evaluation of the outcome indicators; (6) the adequacy of the follow-up time; (7) the loss rate was less than 5%; (8) whether the sample size was estimated; (9) the appropriateness of the selection of the control group; (10) the synchronization of the control group; (11) the comparability of the baseline between groups; (12) the appropriateness of the statistical analysis; NA, not applicable.

**TABLE 4:** CSS quality assessment results.

Included studies	(1)	(2)	(3)	(4)	(5)	(6)	(7)	(8)	(9)
Voss et al., 2011	①	①	③	③	①	③	①	③	③
Chaddock et al., 2012	①	①	③	③	①	①	①	③	③
Herting and Nagel, 2013	①	①	③	③	①	①	①	③	③

(1) Is the source of the information specified? (2) Are inclusion and exclusion criteria for the exposed and non-exposed groups listed or reference to previous publications? (3) Is the time period for identifying patients given? (4) Do subjective factors of the evaluator obscure other aspects of the study population? (5) Describes any assessment for quality assurance; (6) Explains the rationale for excluding any patients from the analysis; (7) Describes how confounding factors were evaluated and/or measures to control for confounding; (8) If possible, explains how missing data were handled in the analysis; (9) Summarizes the response rate of patients and the completeness of data collection; ①, yes; ②, no; ③, unclear.

### 3.4 Frequency analysis of exercise induced activation of brain areas

**FIGURE 2:**
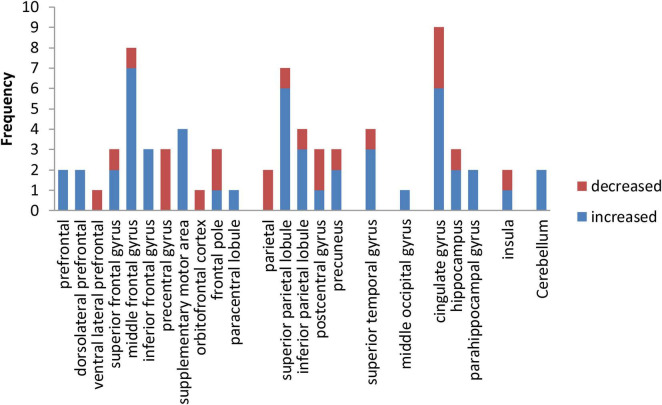
Frequency distribution of exercise-induced increased and decreased activation of brain areas in children and adolescents.

In this study, frequency analysis of brain area activation outcomes was performed based on two dimensions of exercise-induced increased and decreased brain activation in children and adolescents. The results (Figure 2) showed that exercise induced increased or decreased activation in seven macroscopic brain areas, namely the frontal, parietal, temporal, occipital, limbic system, insula, and cerebellum, involving 23 brain areas. Specifically, exercise altered the activation status of the prefrontal, dorsolateral prefrontal, ventral lateral prefrontal, superior frontal gyrus, middle frontal gyrus, inferior frontal gyrus, precentral gyrus, supplementary motor area, orbitofrontal cortex, frontal pole and paracentral lobule in the frontal region; the parietal, superior parietal lobule, inferior parietal lobule, postcentral gyrus, and precuneus in the parietal region; the superior temporal gyrus in the temporal region; the middle occipital gyrus in the occipital region; and the cingulate gyrus, hippocampus, and parahippocampal gyrus in the limbic system.

In addition, this study performed a χ^2^ test for the frequency of increased and decreased activation in macroscopic brain areas. Exercise induced increased activation in the occipital region and cerebellum, so it was not convenient to perform statistical analysis in this study. The results (Table 5) show that the frequency of exercise-evoked increased activation in the frontal (χ^2^ = 10.093, *P* = 0.001) and limbic system (χ^2^ = 5.145, *P* = 0.023) was significantly higher than the frequency of decreased activation.

**TABLE 5:** Results of the χ2 test for exercise-induced changes in the activation status of macroscopic brain areas in children and adolescents.

Brain areas	Activate increased frequency	Activate decreased frequency	χ^2^	*P*
Frontal	22	9	10.093	0.001
Parietal	12	7	2.632	0.105
Temporal	3	1	2.000	0.157
Limbic system	10	4	5.145	0.023
Insula	1	1	0.000	1.000

### 3.5 ALE meta-analysis results

**FIGURE 3:**
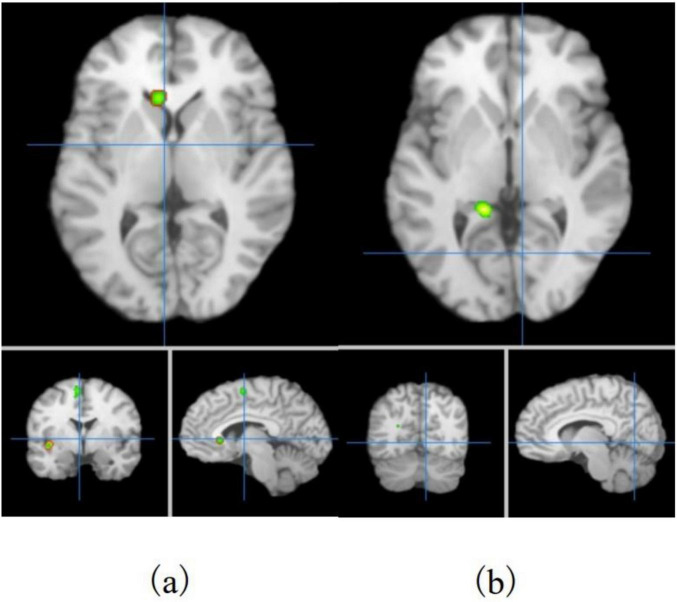
Distribution of exercise-induced increased **(a)** and decreased **(b)** activation brain areas in children and adolescents.

In this study, ALE meta-analysis was performed based on two dimensions of exercise-evoked increased and decreased brain activation, and the results (Table 6 and Figure 3) showed that the exercise intervention obtained six peak activation sites distributed on the left side of the brain, including four peak activation increased sites and two peak activation decreased sites, with a total volume of 2576 m^3^ in the former activated brain area and 752 m^3^ in the latter activated brain area. The four exercise- evoked clusters of increased activation included (1) brain areas dominated by the left medial frontal gyrus (89.7%), paracentral lobule (5.9%), and cingulate gyrus (4.4%) (BA6/31, X = 0, Y = 0, Z = 62); (2) brain areas dominated by the left precentral gyrus (56.3%) and middle frontal gyrus (43.8%) (BA8/9, X = −34, Y = 24, Z = 34); (3) brain areas dominated by the left middle frontal gyrus (85.7%) and inferior frontal gyrus (14.3%) (BA9/46, X = −46, Y = 24, Z = 24); and (4) left caudate nucleus (X = −6, Y = 22, Z = 2). The two clusters of exercise-evoked decreased activation included (1) brain areas on the left side dominated by the parahippocampal gyrus (73.8%), cerebellar apex (14.3%) and lingual gyrus (11.9%) (BA19/27/30, X = −14, Y = −42, Z = 0); (2) brain areas on the left side dominated by the posterior cingulate gyrus (52.0%) and cuneus (48.0%) (BA18/30/ 31, X = −22, Y = −68, Z = 18). In addition, in the MACM results (Table 7), this study found that the activation region dominated by the left intrafrontal gyrus overlapped with the brain function activation map in the NeuroSynth program (*Z* = 33.11), indicating that exercise can induce the enhancement of functional activation closely related to cognition.

**TABLE 6:** Activation cluster results from ALE meta-analysis.

Activation	Brain areas	BA areas	Hemisphere	Volume/m^3^	Talairach Coordinate			ALE value ( × 10^–2^)
					**X**	**Y**	**Z**	
Increased	89.7% intrafrontal gyrus / 5.9% paracentral lobule / 4.4% cingulate gyrus	BA6/31	Left	1064	0	0	62	1.11
	56.3% precentral gyrus/43.8% middle frontal gyrus	BA8/9	Left	616	−34	24	34	1.49
	caudate nucleus	caudate nucleus	Left	472	−6	22	2	1.27
	85.7% middle frontal gyrus/14.3% inferior frontal gyrus	BA9/46	Left	424	−46	24	24	1.15
Decreased	73.8% parahippocampal gyrus/14.3% cerebellar apex /11.9% lingual gyrus	BA19/27/30	Left	544	−14	−42	0	1.14
	52.0% posterior cingulate gyrus/48.0% cuneus	BA18/30/31	Left	208	−22	−68	18	0.94

**TABLE 7:** The results of co-activation maps.

Brain areas	Hemisphere	Talairach Coordinate			*Z*-score
		**X**	**Y**	**Z**	
89.7% intrafrontal gyrus / 5.9% paracentral lobule / 4.4% cingulate gyrus	Left	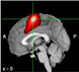	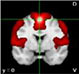	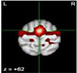	33.11
56.3% precentral gyrus/43.8% middle frontal gyrus	Left	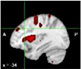	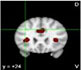	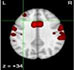	0.00
caudate nucleus	Left	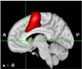	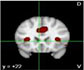	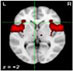	0.00
85.7% middle frontal gyrus/14.3% inferior frontal gyrus	Left	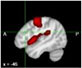	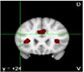	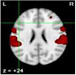	0.00
73.8% parahippocampal gyrus/14.3% cerebellar apex /11.9% lingual gyrus	Left	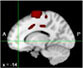	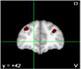	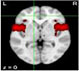	0.00
52.0% posterior cingulate gyrus/48.0% cuneus	Left	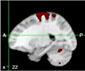	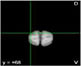	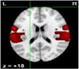	0.00

### 3.6 The test of moderator variables

**FIGURE 4:**
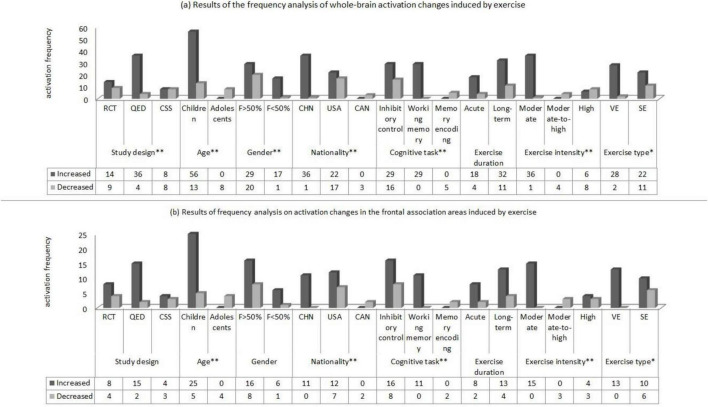
Results of frequency analysis on activation changes in the whole brain **(a)** and frontal association areas **(b)** of children and adolescents induced by exercise (Notes and Abbreviations: RCT, randomized controlled trial; QED, quasi-experimental design; CSS, cross-sectional study; F>50% indicates that the proportion of female participants exceeds 50%; F<50% indicates that the proportion of female participants is below 50%; CHN, China; USA, United States; CAN, Canada; VE, variable exercise; SE, sustained exercise; * indicates that *P* < 0.05; ** indicates that *P* < 0.01).

The results of frequency analysis of exercise-induced brain activation in children and adolescents (Figure 4) showed that: (1) The increased and decreased of whole- brain activation induced by different study designs were statistically significant (*P* < 0.01), among which the brain regions with enhanced activation in the QED experiment were significantly more than those with decreased activation; (2) The increased and decreased of whole-brain and frontal association area activation induced by different ages were statistically significant (*P* < 0.01), among which the brain regions with increased activation in whole-brain and frontal association area in children were significantly more than those with decreased activation; (3) The increased and decreased of whole-brain activation induced by different genders were statistically significant (*P* < 0.01), among which the brain regions with increased whole-brain activation induced by experiments with less than 50% female participants were significantly more than those with decreased activation; (4) The increased and decreased of whole-brain and frontal association area activation induced by different nationalities were statistically significant (*P* < 0.01), among which the brain regions with increased activation in whole-brain and frontal association area of Chinese participants were significantly more than those with decreased activation; (5) The increased and decreased of whole-brain and frontal association area activation induced by different cognitive tasks were statistically significant (*P* < 0.01), among which the brain regions with increased activation in whole-brain and frontal association area induced by working memory tasks were significantly more than those with decreased activation; (6) The increased and decreased of whole-brain and frontal association area activation induced by exercise intensity were statistically significant (*P* < 0.01), among which the brain regions with increased activation in whole-brain and frontal association area induced by moderate-intensity exercise were significantly more than those with decreased activation; (7) The increased and decreased of whole- brain and frontal association area activation induced by exercise types were statistically significant (*P* < 0.05), among which the brain regions with increased activation in whole-brain and frontal association area induced by variable exercise were significantly more than those with decreased activation. In addition, this study has not found that the increased and decreased of whole-brain and frontal association area activation induced by exercise duration were statistically significant (*P* > 0.05).

**FIGURE 5:**
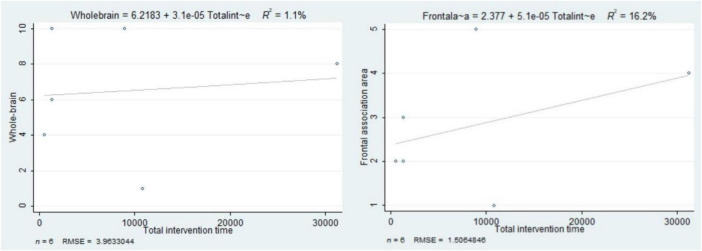
The association between total intervention time and the number of activated brain regions in the whole brain and frontal association areas.

The total intervention time contributed to 1.1% of the variability in the number of activated brain regions in the whole-brain during exercise interventions, and 16.2% of the variability in the frontal association areas (Figure 5). In other words, the longer the total intervention time, the greater the number of activated brain regions.

This study focuses on analyzing the differences in brain region activation induced by sustained and variable exercise. The results (Table 8) show that continuous exercise induced decreased activation in the left side of the brain areas dominated by the parahippocampal gyrus (50.0%), culmen (39.1%) and lingual gyrus (10.9%) (BA19/27/30, X = −14, Y = −42, Z = 0), with a volume of 840 m^3^ of activated brain areas, while variable exercise induced increased activation in the left middle frontal gyrus (BA46, X = −46, Y = 24, Z = 24), with a volume of 592 m^3^ of activated brain areas.

**TABLE 8:** ALE meta-analysis activation cluster results for different exercise types.

Exercise types	Activation	Brain areas	BA areas	Hemisphere	Volume/m^3^	Talairach Coordinate			ALE value ( × 10^−2^)
						**X**	**Y**	**Z**	
Sustained exercise	Decreased	50.0% parahippocampal paracentral gyrus/39.1% culmen/10.9% lingual gyrus	BA19/27/30	Left	840	−14	−42	0	1.14
Variable exercise	Increased	Middle frontal gyrus	BA46	left	592	−46	24	24	1.15

## 4 Discussion

### 4.1 Frontal association area dominate in exercise-induced brain activation

Based on the results of ALE meta-analysis, this study found increased and decreased exercise-induced brain activation and presence in the fMRI series of experiments, and the activation effect was more prominent in the frontal association area. Exercise altered the activation status of a series of brain regions in the frontal association area (intrafrontal gyrus, middle frontal gyrus, inferior frontal gyrus and precentral gyrus), parietal (cuneus), occipital (lingual gyrus), limbic system (cingulate gyrus and parahippocampal gyrus), striatum (caudate nucleus) and cerebellum. The activation volume of the frontal association area was 2057 m^3^, accounting for 61.81% of the activation cluster volume. In addition, the activation of frontal association area showed increased effects, thus the results support the “extended frontoparietal integration theory” and the “neural efficiency hypothesis”. Since the improvement of cognitive or behavioral tasks in humans is the result of the synergistic action of neurons in multiple brain areas (Popescu et al., 2009), the improvement of cognition or behavior due to exercise is also necessarily the result of improved functional connectivity in multiple brain areas. In addition, the results of the brain network analysis by Chen et al. (2016a) showed a significant positive correlation between the functional connectivity of the right inferior frontal gyrus-right cerebellum and 2-back task performance; Chen et al. (2017) showed that the functional connectivity of the right dorsolateral prefrontal-left cerebellum was significantly and negatively correlated with Flanker task performance. The above study further supports the results of our study.

Exercise-induced brain plasticity does not manifest only as a single increased or decreased activation, but rather as a coexistence of increased and decreased activation. The simultaneous presence of exercise-induced increased and decreased activation in brain areas in part suggests that exercise improves the efficiency of coordinated central nervous processing (Genç et al., 2018; Guo et al., 2017). The dual processing model of cognitive control (Schneider and Shiffrin, 1977; Wei, 2019) states that acquired exercise can facilitate a shift from controlled processing that requires consciousness to automated processing that does not require specialized control, reducing the use of individual conscious resources and conserving central attention resources. Among the activation-enhancing brain areas, the activation in the frontal association area was more intensive. The frontal lobe is the most developed brain lobe in the body and is involved in higher cognitive functions such as language processing, number crunching, and working memory (Boisgueheneuc et al., 2006; Koyama et al., 2017) as well as playing a regulatory role in decision making and execution (Rushworth et al., 2004). Long-term exercise improves people’s language processing and reading fluency (Scudder et al., 2014), number crunching and logical thinking (Haapala et al., 2017; Howie et al., 2015), executive function and academic performance (Fu and Fan, 2016), and these behavioral-level changes somewhat support the results of brain activation. In addition, groups that engage in long-term exercise have higher self-control (Fu and Liu, 2018; Yang, 2022) and a good self-control is important for planning and problem solving as well as program execution. However, exercise induces decreased activation of the parahippocampal gyrus and posterior cingulate gyrus during cognitive tasks in children and adolescents. The cingulate gyrus and the fronto-parietal association area are thought to scaffold human cognitive and thinking activities (Schneiders et al., 2011) and are involved in functions such as emotion, learning, and memory; the parahippocampal gyrus plays an important role in cognitive tasks such as spatial memory and navigation (Bohbot et al., 2015), and its decreased activation may be relevant to the fMRI experimental task. The cognitive task paradigm involved in their study hardly involves complex spatial memory extraction and storage, and therefore does not reflect the main functions of the above-mentioned brain areas.

### 4.2 The moderating variables of exercise-induced brain plasticity

#### 4.2.1 Study design

This study found that in QED, the number of brain regions showing increased activation induced by exercise was significantly greater than the number showing decreased activation, indicating that the research design may have caused heterogeneity in the results of brain region activation. However, QED lacks random assignment, which cannot fully control research conditions, and there may be selection bias and other systematic biases, resulting in more interference from external factors and affecting the accuracy of causal inference. Therefore, it is recommended that future studies adopt more RCTs to explore the brain plasticity induced by exercise in children and adolescents, in order to improve the reliability of research results.

#### 4.2.2 Characteristics of participants

Firstly, the number of brain regions activated by exercise in children is significantly greater than the number of those deactivated, and this result is not observed in adolescents. In terms of the development of executive control functions in the brain, Wang and Su (2006) have found that individuals generally reach adult levels in many standardized executive function tests around age 12. Zhao et al. (2015) have shown that younger children have a more significant advantage in terms of cognitive improvement induced by exercise, suggesting that exercise is more likely to induce brain plasticity in younger children. Furthermore, Wang et al. (2019) have also further confirmed this finding, revealing that exercise has positive promoting benefits for children, adolescents, and the elderly, but the effect size in the child population is significantly higher than that in other groups.

Secondly, in studies where girls make up less than 50%, exercise induces more activation enhancement in brain regions, while this result has not yet been found in studies where girls are less than 50%. The maturity of various regions of the female brain reaches its peak 2 to 4 years earlier than that of boys (Sabbatini, 1997), thus typically requiring more time for intervention to achieve positive benefits (Zhao et al., 2015). In addition, gender roles and expectations may affect an individual’s psychological response to exercise. Sociocultural tendencies to encourage boys to participate more in exercise may lead to boys having a more positive psychological expectation for exercise, which could enhance brain activation after exercise.

Finally, this study found that exercise induced a significant increase in the number of activated brain regions among Chinese children and adolescents, with more activations than deactivations. This study has not yet found research on the differences in brain region activation induced by exercise among participants of different nationalities or ethnicities. The explanation for this finding may involve cultural differences, education systems, lifestyles, sample selection, research methods, and other aspects, but further research is needed to determine the specific reasons.

#### 4.2.3 Cognitive task

This study found that exercise significantly increased the number of brain regions activated in the whole brain and frontal lobe association areas during working memory tasks, compared to a decrease. However, this finding was not reflected in inhibitory control and memory encoding tasks. Firstly, inhibitory control and working memory are sub-components of executive function. The former is the process of purposefully controlling an individual’s habitual responses, while the latter is the process of managing and decoding new information in the cognitive process, while replacing old irrelevant information with new information (Shi et al., 2022). Therefore, in terms of task difficulty, working memory tasks are significantly more challenging than inhibitory control tasks, thus requiring a broader range of cognitive resources during working memory tasks. Secondly, only one study (Herting and Nagel, 2013) has used memory encoding tasks for research, which may limit the accuracy of the results, and we look forward to further exploration in subsequent studies.

#### 4.2.4 Characteristics of exercise

Firstly, the number of brain regions activated by moderate-intensity exercise significantly exceeds the number of regions with reduced activation, a finding similar to previous studies exploring the relationship between exercise intensity and cognitive performance (Xie, 2020; Shi et al., 2022). For example, Xie (2020) compared the intervention effects of moderate and high-intensity exercise on children and adolescents’ working memory, finding that moderate-intensity exercise has a positive intervention effect on both children and adolescents, while the intervention effect of high-intensity exercise is not significant, which may be related to the excessive fatigue induced by high-intensity exercise. Shi et al. (2022), based on a real exercise scenario, also presented similar results, showing that both acute and long-term interventions of moderate exercise are conducive to improving the executive functions of children and adolescents.

Secondly, although there is no statistical difference in the number of brain regions activated or deactivated by acute and long-term exercise, in long-term interventions, the longer the total intervention time, the more activation in the frontal association areas. Long-term exercise may promote the neuroplasticity of the brain, that is, the adaptive changes of the brain’s structure and function to experience. The frontal lobe association areas are regions in the brain related to executive functions, decision-making, and complex cognitive tasks. Long-term exercise may enhance the neural connections and the efficiency of neural networks in these areas, thereby increasing their activation (Chaddock-Heyman et al., 2013). In addition, long-term exercise may be accompanied by individuals’ psychological adaptation and habit formation to exercise, and this change in psychological state may affect brain activity, especially in the frontal lobe areas related to emotional regulation and motivation (Shi et al., 2023).

Finally, exercise-evoked brain plasticity during cognitive tasks in children and adolescents differed in exercise types, that is, sustained exercise evoked decreased activation of the parahippocampal gyrus, Culmen and lingual gyrus, whereas variable exercise evoked increased activation of the frontal middle gyrus. For sustained exercise, the results of this study were similar to a previous study (Metcalfe et al., 2016). Their findings showed that a single moderate-to-vigorous intensity power bike intervention induced decreased activation of the parahippocampal gyrus. Activation of the parahippocampal gyrus may be related to the exercise types chosen by the subjects; subjects who engage in long-term variable exercise such as basketball may have higher brain white matter volume in the parahippocampal gyrus areas (Shen et al., 2014), while participants who engage in long-term closed skill exercise such as diving have lower brain gray matter density in this area (Wei et al., 2009). In addition, the Culmen area of the cerebellum is important for maintaining body balance and coordination; while the lingual gyrus is responsible for receiving visual information and is involved in analyzing complex images and storing visual memories (Mendoza et al., 2011). Although Culmen and the lingual gyrus have some control over cognitive and thinking activities, this modulation is not as strong as in the frontal association area; therefore, the decreased activation in the above mentioned areas is a reflection of neural efficiency. For example, Wei et al. (2009) based on a voxel-based method of morphological analysis of the whole brain showed that the gray matter volumes in the right cerebellum and left lingual gyrus were much smaller in divers than in the average participants; Guo et al. (2017) compared the differences in brain area activation between table tennis players and normal participants when they were stimulated with the GO/NO GO task based on fMRI, and found that table tennis players had significantly decreased activation in the bilateral lingual gyrus. For variable exercise, the results of this study are similar to the results of previous ALE meta-analyses (Lou and Liu, 2020) on the activation state of the athlete’s brain. The study included athletes engaged in open skills training in basketball, soccer, volleyball, tennis, badminton and field hockey and found that athletes had stronger activation in the middle frontal gyrus than controls in all cases. Thus, variable exercise is more conducive to plasticity in the frontal association area, which in turn is more conducive to promoting cognitive brain function benefits in participants.

### 4.3 Limitations of this study

Firstly, due to the limitation of search language, this study primarily searched for research in Chinese and English, potentially omitting relevant research findings that were published in other languages. Secondly, considering the limited number of original RCTs, this study included QEDs and CSSs in the ALE meta-analysis, which may interfere with the accuracy of the study results. Particularly in QEDs, there may be selection bias and systematic errors due to the lack of random assignment and the inability to fully control research conditions. Lastly, given the limited number of original studies, although this study explored relevant moderating variables, the number of original studies in some subgroups is relatively small, thus the results of this study should be interpreted with caution.

### 4.4 Implications for exercise intervention practice

This study found that variable exercise can induce more activation in the frontal associative areas, which serves as a reminder that in intervention practices, we should pay more attention to the moderating role of exercise types. Interventions should incorporate elements that promote variability to enhance the brain function of children and adolescents. Firstly, by adding key elements that promote variability in practice to the intervention, including environmental stimuli, interpersonal interactions, and complex tasks, the aim is to trigger cognitive challenges in children and adolescents and to enhance the activation of the frontal association area. In addition, it is becoming the norm for students in China to learn options based on their sport interests, and the benefits of different types of skills on brain plasticity and cognitive facilitation are different. Therefore, it is recommended that sports skill instruction incorporate the above elements into the design of learning and practice activities. By optimizing the teaching context, improving the teaching atmosphere, and providing complex tasks, both exercise interests are satisfied and the plasticity of the frontal association area is increased. Secondly, exercise methods that incorporate motor-cognitive dual-tasks can be adopted to improve the allocation of cognitive resources in children and adolescents during exercise and enhance the brain’s engagement in cognitive tasks. The so-called motor-cognitive dual-task exercise refers to the form of exercise where cognitive, thinking, and decision-making tasks are performed during exercise. This type of exercise shares similarities with variable exercise, in that it involves cognitive participation amidst sustained repetitive movements, which may have a positive effect on promoting brain plasticity. Finally, exercise based on digital screens like Xbox shares similarities with variable exercise; the former offers a rich array of games that involve cognitive challenges, which has a positive significance for shaping the prefrontal cortex.

## 5 Conclusion

Exercise promotes improved brain cognitive task performance by inducing plasticity in the frontal association area. In view of the general characteristics of exercise-induced brain plasticity, this study was based on the “extended frontoparietal integration theory” and the “neural efficiency hypothesis”, and found that the activation volume of the frontal association area accounted for 61.81% of the activation cluster volume induced by exercise based on ALE analysis. This study confirms that the frontal association area is a key site of exercise-induced brain plasticity. Additionally, the increase and decrease in the number of brain region activations induced by exercise exhibit differences in study design, age, gender, nationality, cognitive tasks, exercise intensity, duration, and type of exercise. Particularly, it was found that exercise induces differences in brain plasticity in children and adolescents based on the type of exercise. That is, sustained exercise induced a decrease in activation in the left parahippocampal gyrus, culmen, and lingual gyrus, while variable exercise induced an increase in activation in the left middle frontal gyrus. For exercise interventions, this study emphasizes that longer intervention times with moderate-intensity variable exercise can induce greater brain plasticity and exert a more active cognitive enhancement benefit.

## Data Availability

The raw data supporting the conclusions of this article will be made available by the authors, without undue reservation.

## Footnotes

1
http://www.brainmap.org/ale/

2
http://NeuroSynth.org/
