# Supplementary material for: The frontal association area: exercise-induced brain plasticity in children and adolescents and implications for cognitive intervention practice
Source: Front Hum Neurosci. 2024 Sep 5;18:1418803. doi: 10.3389/fnhum.2024.1418803 (PMC11410640; doi:10.3389/fnhum.2024.1418803)
Supplement: Supplementary file 1 [file Data_Sheet_1.docx]

# Supplementary Material 1: Changes in brain activation results

| Included studies | Increased | Decreased |
| --- | --- | --- |
| Chen et al., 2011 | Right superior frontal gyrus  Right middle frontal gyrus  Right superior parietal lobule  Left middle frontal gyrus  Left superior frontal gyrus  Left superior parietal lobule  Left inferior parietal lobule | Left anterior cingulate gyrus |
| Davis et al., 2011 | Left prefrontal cortex  Right prefrontal cortex | Left posterior parietal cortex  Right posterior parietal cortex |
| Voss et al., 2011 | Left postcentral gyrus  Left insular cortex  Left middle frontal gyrus | Left postcentral gyrus  Left insular cortex  Left middle frontal gyrus |
| Chaddock et al., 2012 | Left middle frontal gyrus  Right middle frontal gyrus  Left supplementary motor area  Right anterior cingulate gyrus  Left superior parietal cortex |  |
| Chaddock-Heyman et al., 2013 |  | Right frontal pole |
| Herting et al., 2013 |  | Left superior temporal gyrus  Left ventromedial prefrontal cortex  Right posterior cingulate gyrus  Right inferior parietal lobule  Right superior frontal gyrus |
| Krafft et al., 2014 | Left superior temporal gyrus  Left medial frontal gyrus  Right anterior cingulate gyrus  Left middle frontal gyrus | Right precentral gyrus  Left precentral gyrus  Right postcentral gyrus  Right superior parietal lobule  Left precentral gyrus |
| Jin, 2016 | Left parahippocampal gyrus  Right parahippocampal gyrus  Left precuneus  Right precuneus  Left posterior cingulate gyrus  Right posterior cingulate gyrus  Left superior temporal gyrus  Right dorsolateral prefrontal cortex  Left dorsolateral prefrontal cortex  Left frontal pole |  |
| Chen et al., 2016 | Left cerebellar hemisphere posterior lobe  Bilateral cerebellar hemisphere posterior lobes  Left middle frontal gyrus  Right anterior cingulate gyrus  Right medial frontal gyrus  Right middle frontal gyrus  Right superior occipital gyrus  Right posterior cingulate gyrus  Left middle frontal gyrus  Left superior parietal lobule  Right inferior parietal lobule |  |
| Metcalfe et al., 2016 |  | Left orbital frontal cortex  Right frontal pole  Bilateral hippocampal regions |
| Sachs et al., 2017 | Left supplementary motor area  Right supplementary motor area  Left anterior cingulate gyrus  Right anterior cingulate gyrus  Left precentral gyrus  Left anterior insula  Left inferior frontal gyrus  Left intraparietal sulcus |  |
| Zhu et al., 2021 | Left inferior frontal gyrus  Right middle occipital gyrus  Left hippocampus  Left superior temporal gyrus  Left middle frontal gyrus  Right inferior parietal lobule |  |
| Qu et al., 2024 | Left supplementary motor area  Left paracentral lobule |  |

# Supplementary Material 2: A narrative review of exercise-induced brain plasticity

### 1 Exercise induces increased, decreased, or re-organized brain activation under inhibitory control tasks

The Flanker task is a classic paradigm for the study of executive function interference inhibition by causing interference in participants’ judgments of central target stimuli through bilaterally distracting stimuli. A single 30-min moderate-intensity power bike intervention induced increased activation in the frontoparietal association area and decreased activation in the anterior cingulate gyrus in children (Chen et al., 2011). Long-term moderate to vigorous intensity aerobic exercise induces increased activation of the internal frontal gyrus, middle frontal gyrus, anterior cingulate gyrus, and superior temporal gyrus in children (Krafft et al., 2014). The results of cross-sectional studies are inconsistent. Chaddock et al. (2012) found that children with high physical fitness exhibited more activation in the frontoparietal association area; whereas Voss et al. (2011) found that brain activation in children with high physical fitness underwent re-organized. In addition, the Stroop task requires subjects to judge the color of words as quickly and correctly as possible, regardless of their names and meanings, and is a research paradigm that reflects interference inhibition. Long-term combined soccer- and swimming-led exercise induces increased activation of brain areas in the frontal inferior gyrus, anterior cingulate gyrus, and supplementary motor cortex in children (Sachs et al., 2017).

The GO/NO GO task, which requires participants to make judgments about two different stimuli that alternate randomly and respond to one of them (GO response) and not to the other (NO GO response), is the classic paradigm for studying response inhibition. Acute power bike interventions induce decreased activation in areas such as the frontal pole, orbitofrontal cortex and hippocampus in adolescents (Metcalfe et al., 2016); whereas long-term comprehensive interventions aimed at improving cardiorespiratory fitness, muscle strength and motor skills induce decreased activation in the prefrontal and anterior cingulate gyrus in children (Chaddock-Heyman et al., 2013). In addition, the Antisaccade task, which requires subjects to inhibit gaze at a peripheral stimulus target and gaze at its opposite, is a research paradigm for response inhibition. Long-term moderate to vigorous intensity aerobic exercise induces decreased activation of the somatosensory motor area, anterior cingulate gyrus and superior parietal lobule during the Antisaccade task in children (Krafft et al., 2014). A moderate-intensity comprehensive exercise intervention consisting of running games, jump rope, basketball, and soccer induces increased activation in the prefrontal lobe and decreased activation in the posterior parietal lobe in children (Davis et al., 2011).

### 2 Exercise-induced increased brain activation under working memory tasks

The N-back task requires participants to compare the current stimulus with the previous Nth stimulus; when N=1, subjects are asked to compare the current stimulus with the previous stimulus adjacent to it; when N=2, they compare the current stimulus with the stimulus one position away from it; and so on to obtain different levels of task difficulty, which is the classic research paradigm for working memory refreshment. Exercise induces increased brain activation under the N-back task in children. Specifically, a 30-min moderate-intensity power bike intervention promoted increased activation in the parietal lobe, hippocampus, and cerebellum (Chen et al., 2016); an 11-week moderate-intensity combination exercise program dominated by pattern running, martial arts exercises, and fancy jump rope was effective in promoting increased activation in the frontoparietal association area, superior temporal gyrus, precuneus, and parahippocampal gyrus (Jin, 2016; Qu et al., 2024); and an 11-week leisure exercise dominated by running games, rope skipping, and martial arts promoted increased activation in the frontoparietal association area, middle occipital gyrus, superior temporal gyrus, and hippocampus (Zhu et al., 2021).

### 3 Exercise induces decreased brain activation under associative memory task

The lexical associative memory encoding task requires subjects to make judgments about the matching of presented words and is a research paradigm for memory recognition tasks. Herting et al. (2013) showed that the high fitness group evoked decreased activation of the frontoparietal association area and superior temporal gyrus compared to the low fitness group, although they did not present a higher memory advantage. That is, high-fitness adolescents use fewer neural resources to achieve the same task performance.

### 4 Brief conclusions

In conclusion, exercise induces activation of the functional association areas of the task state brain, and the involvement of more brain areas also supports the neuroplasticity of exercise, to some extent reflecting the whole brain functional association. Furthermore, exercise-induced increase and decrease in activation of brain areas co-exist in existing fMRI studies, which may be associated with specific exercise intervention protocols and cognitive task paradigms, among others.
